# Culture and conservation in baleen whales

**DOI:** 10.1098/rstb.2024.0133

**Published:** 2025-05-01

**Authors:** Ellen C. Garland, Peter Corkeron, Michael J. Noad, Briana Abrahms, Jenny A. Allen, Rochelle Constantine, Luke Rendell, Renata S. Sousa-Lima, Kathleen M. Stafford, Emma L. Carroll

**Affiliations:** ^1^ Centre for Social Learning and Cognitive Evolution, University of St Andrews, St Andrews, UK; ^2^ Sea Mammal Research Unit, School of Biology, University of St Andrews, St Andrews, UK; ^3^ Centre for Planetary Health and Food Security, Griffith University, Nathan, QLD, Australia; ^4^ Cetacean Ecology and Acoustics Laboratory, School of Veterinary Science, The University of Queensland, Brisbane, QLD, Australia; ^5^ Center for Ecosystem Sentinels, Department of Biology, University of Washington, Seattle, WA, USA; ^6^ Bio-Telemetry and Behavioral Ecology Laboratory, Institute of Marine Science, Long Marine Laboratory, University of California Santa Cruz, Santa Cruz, CA, USA; ^7^ Southern Ocean Persistent Organic Pollutants Programs (SOPOPP), Griffith University, Nathan, QLD, Australia; ^8^ School of Biological Sciences, The University of Auckland – Waipapa Taumata Rau, Auckland, New Zealand; ^9^ Graduate Program of Psychobiology and Behavior, Biosciences Center, Universidade Federal do Rio Grande do Norte, Natal, Rio Grande do Norte, Brazil; ^10^ Laboratory of Bioacoustics, Department of Physiology and Behavior, Biosciences Center, Universidade Federal do Rio Grande do Norte, Natal, Rio Grande do Norte, Brazil; ^11^ Marine Mammal Institute, Oregon State University, Newport, OR, USA

**Keywords:** cetacean, animal culture, migratory culture, foraging traditions, vocal dialects, conservation management

## Abstract

Culture, defined as information or behaviours shared within a population and acquired from conspecifics through social learning, acts as a second inheritance system which has important implications for species' ecology and evolution. Understanding the influence of social learning and culture in animals' lives is essential to planning and predicting outcomes of conservation actions. Culture plays a key role in cetaceans’ lives, and some of the best evidence for social learning has come from baleen whales (Mysticetes). The Convention on the Conservation of Migratory Species of Wild Animals (CMS), an international treaty operating under the United Nations Environment Programme (UNEP), has recently integrated animal culture into their conservation initiatives. An essential first step in such a conservation framework is to identify key indicators of social learning in well-studied species, which could be applied to other more elusive species. Here, as part of the UNEP CMS Expert Group on Animal Culture and Social Complexity, we first evaluate key evidence for social learning and culture in baleen whales through several case studies. We then suggest key indicators by behavioural context to assist in identifying potential cases of social learning in more elusive species generating a practical guide for future conservation assessment and management.

This article is part of the theme issue ‘Animal culture: conservation in a changing world’.

## 1. Introduction

Conservation approaches and decision-making have the key goal of maintaining genetic and phenotypic diversity to ensure long-term persistence and adaptive potential of populations [1]. One phenotypic difference within and among populations can be behaviour [2,3]. Behavioural differences often arise and are maintained through social learning [4]. This has led to the increasing recognition of social learning and resultant culture as important factors that may impact the viability and structure of populations [5,6]. ‘Social learning’ is defined here as any learning process facilitated by the observation of, or interaction with, another conspecific or its products [6–9]. Information and/or behaviours acquired through social learning can flow in various directions: vertically from parents to offspring, obliquely from older often unrelated individuals to younger individuals and horizontally between peers [10].

From the building blocks of social learning, information may be transmitted through populations, creating local behaviours that persist over generations and time as ‘traditions’ [6]. ‘Culture’ is defined here, following others, as information or behaviours shared within a population and acquired from conspecifics by some form of social learning [5–7,11,12]. Cultures can therefore include traditions, especially those that involve a large proportion of a population, as well as rapidly changing behaviours that are modified by social learning. Social learning and culture can occur across a diversity of contexts, from mate choice to foraging to migration routes [13–16] and multiple cultural traits may be present within a population or species [17]. For example, humpback whales (*Megaptera novaeangliae*) can maintain multiple independently evolving cultural traits within a population as they display song (vocal) culture, migratory culture and socially learned foraging traditions [17–20]. These baleen whales are one of the best studied cetaceans, but many others also display various socially learned behaviours (e.g. foraging traditions in bottlenose dolphins (*Tursiops* spp.), shared vocal repertoires and feeding traditions in killer whales (*Orcinus orca*) and vocal clans and cultural hitchhiking in sperm whales (*Physeter macrocephalus*) [7,21,22]. Importantly, baleen whale calves often spend the first year of their lives with their mother, creating an opportunity for social learning through vertical transmission of behaviours [16,18]. Additionally, there is evidence of repeated learning of new behaviours beyond these early years (e.g. song learning [23,24]) facilitated by horizontal transmission.

Here, we explore different case studies of social learning in baleen whales by behavioural context with the aim of assessing how broadly social learning may operate in these taxa and how such behaviours could interface with conservation management [6]. Case studies were chosen based on their ability to present the best available evidence in baleen whales of social learning by behavioural context. Previous publications [5,6,25,26], and other articles in this theme issue, make the case that social learning and animal culture have important implications for conservation [27] through the interface of socially learned behaviour with vital rates, habitat use and responses to conservation interventions [28–30]. Social learning can be a cause, consequence or marker of demography, vital rates, population genetic structure, phenotypic diversity and ecological niche separation [6,31,32]. We acknowledge that ‘gold standard’ evidence involving controlled field or laboratory experiments is not always possible or potentially unethical in endangered and elusive species such as baleen whales. Instead, we rely on observed (or correlative) patterns of behavioural expression to infer the presence of cultural processes and apply the precautionary principle [6,33]. Specifically, we use case studies to elucidate if social learning could influence foraging, migration and vocal behaviour in baleen whales, and then consider the wider conservation implications of such behaviour.

## 2. Foraging behaviours and traditions

### (a) Socially learned foraging tactics in humpback whales

Baleen whales exhibit a range of foraging tactics, but little is known of their development. A notable exception is the spread of ‘lobtail feeding’ observed among the Gulf of Maine humpback whales in the USA [17]. This is a behavioural sequence consisting of an individual hitting the water’s surface with the fluke several times (known as ‘lobtailing’), followed by a circular dive in the immediate area during which the individual produces bubbles to form a bubble-net, then a surface lunge within the bubble-net, where the whale gapes and engulfs prey [17,34,35]. The lobtail feeding strategy emerged following a local crash in the herring (*Clupea harengus*) population, and population peaks of sand lance (*Ammodytes americanus*), leading to a shift in prey availability [17]. Over time, the behaviour spread to nearly 40% of the identified population. The strength of the evidence for social transmission in this behaviour is based on several factors. First, the analysis used a large, robust dataset of 653 individuals, each with at least 20 resightings [36]. Second, the study period covered nearly three decades and included the apparent introduction or innovation of lobtail feeding into the population. Finally, when network diffusion models of the lobtail feeding spread included social learning, they were several orders of magnitude better supported than equivalent models without a social learning term [17]. The case of lobtail feeding therefore illustrates strong evidence of horizontal cultural transmission being a key factor in the spread of a foraging behaviour across a population with ecological consequences in terms of prey selection.

Emerging research into animal foraging behaviours suggests an ever-increasing number may be socially learned [6]. In addition to the lobtail feeding example, humpback whales show a high degree of plasticity in their foraging tactics [37], indicating significant potential for additional instances of social learning to emerge from ongoing research. These tactics include lunging [38], the use of bubbles [39,40] or body parts to corral prey [41] or scooping prey from the substrate [42]. Several of these behaviours involve coordination among multiple individuals, making a social component obligatory to their effective use, and perhaps learning. For example, synchronized lunge feeding occurs among large groups on the southeast Alaskan feeding grounds [37], while pairs or small groups of whales in the Gulf of Maine will engage in coordinated bottom-side roll feeding [42,43] and varieties of bubble feeding [44]. Coordination in a few of these strategies is thought to occur through accompanying foraging calls [43]. A specific example of emerging evidence for other socially learned tactics is the ‘trap feeding’ innovation observed off the coast of British Columbia, Canada [41]. This tactic involves an individual remaining at or near the surface with their mouth open and either spinning or using their pectoral fins to push prey towards their mouth [45]. Models of trap feeding suggest that both social associations and residence (which often correlates with social contact) may contribute to the observed spread, both of which factor into cultural transmission. At a wider scale, such population-specific differences in foraging tactics add weight to these behaviours having a socially learned component.

### (b) Bryde’s whale foraging behaviours

Bryde’s whale (*Balaenoptera edeni* spp.) also demonstrates behavioural plasticity in foraging behaviours, prey types and depths within and between regions [46]. We acknowledge the taxonomy of this group is still unclear; therefore, caution is warranted when interpreting behavioural differences as widely separated populations may represent different subspecies or even species. Like other baleen whales, Bryde’s whales typically lunge-feed by engulfing a large volume of water and filtering out prey. However, they can prey switch and change foraging strategy depending on whether they are targeting fish or zooplankton. For instance, in the Hauraki Gulf, Aotearoa/New Zealand, they use a chin-slapping tactic to aggregate zooplankton before rapidly turning on their side to engulf prey. In contrast, the same individuals will switch strategy and lunge at speed directly underneath fish prey [47]. This plasticity of foraging behaviours suggests a social learning component. Records of mothers observed chin-slapping with their calf then lunging through the aggregated prey may indicate vertical transmission of the behaviour [47]. In the Gulf of Thailand, in contrast, Bryde’s whales remain almost stationary at the surface while fish spill into their wide-open mouth. This distinctive and commonly observed behaviour known as ‘tread-water feeding’ has been observed in adults and calves with the calf alongside its mother presumably learning this foraging behaviour [48]. Another population-specific, recently described specialization revealed Bryde’s whales undertaking high-speed chases near the seabed to catch low-density schools of fish in nearshore South African waters [49]. The range of behaviours that Bryde’s whales use, and identification of new local specializations, requires further examination of how novel behaviours eventuate and are transmitted within populations.

## 3. Migratory traditions

### (a) Right whale migratory culture

In the Southern Hemisphere, southern right whales (*Eubalaena australis*) appear to transmit preferences in migratory destinations from mother to offspring in the first year of life [16,50]. Prior to whaling, there were approximately 120,000 southern right whales across 12 wintering grounds [51]. The strength of connection between wintering grounds and mid- to high-latitude Southern Hemisphere foraging areas varied, creating a kind of migratory network [51,52]. Females display strong, long-term site fidelity to specific winter calving grounds [53–55] and have preferred foraging locations. Studies have revealed that southern right whales in Argentinian and Australian waters were significantly more likely to have similar isotope profiles if they had the same mitochondrial DNA haplotype (i.e. maternal lineage) [16,50]. The more related individuals were, the more likely they were to feed in the same location [16]. This suggests learning of foraging grounds from mothers and links the cultural inheritance of migration routes with foraging ecology. When whaling extirpated southern right whales using a specific migratory habitat, this led to the loss of knowledge of that area as a good migratory destination. This loss of ‘cultural memory’, exacerbated by the removal of neighbouring populations and low density, means that once-inhabited areas are unlikely to be recolonized on a timeframe relevant to management (i.e. decades; [56]). This has led to a patchwork of recovery, with some wintering grounds recovering while others remain remnant or potentially extirpated (e.g. Chile–Peru versus mainland New Zealand; see [52]). Such extirpation and loss of cultural memory of migratory habitat has also occurred in other baleen whales, including humpback and blue whales (*Balaenoptera musculus* spp.) from South Georgia, where only very recent recolonization of the area [57] suggests loss of cultural memory for many decades.

This cultural knowledge of migratory habitats appears to shape both southern right whales and North Atlantic right whales (*Eubalaena glacialis*) habitat use and recovery after exploitation. As with southern right whales, North Atlantic right whale calves remain with their mother for around a year and appear to learn foraging sites during this time [58]. Recently, however, the distribution of North Atlantic right whales along the east coast of North America has changed [59], with the species abandoning at least one foraging site. At the same time, North Atlantic right whales have occupied two apparently new foraging sites [60–64]. Similarly, decadal change in foraging ground preferences in multiple southern right whale foraging grounds suggests flexibility [65].

One new North Atlantic right whale foraging site, the Gulf of St Lawrence, is now occupied by a specific subset of individuals, numbering a little under half of the species [64]. These individuals have moved to this alternative feeding location, mostly after 2015 [63]. The sudden change in the movements of a specific cohort of the species is not simple resource tracking. There is very good evidence for social learning of foraging areas, but like many socially learned behaviours, this is plastic and can change; the key here is to better understand the interaction between social learning that creates traditions and social learning (or other processes) that can create relatively quick change in those traditions. Regardless, this behavioural change in foraging ground is of conservation significance. At least 12 whales died in the Gulf of St Lawrence in 2017, as the Canadian government was unaware that this aggregation had appeared. More North Atlantic right whales died in 2019 as managers struggled to quickly address anthropogenic threats (e.g. entanglements in fishing gear and vessel strikes). This contributed to the change in North Atlantic right whales’ IUCN Red List status from Endangered to Critically Endangered. Also, females observed feeding in the Gulf are now more likely to calve than those that do not feed there [66]. As poor reproductive success substantially contributes to their current decline [67], the survival and fecundity of these Gulf-using individuals are imperative (see electronic supplementary material).

### (b) Blue whale migratory memory

Blue whales use spatial memory to enhance the performance of their migrations to foraging grounds [68]. In the eastern North Pacific Ocean, blue whale northward migrations are timed with the spring bloom in prey availability; movements were found to closely match the long-term average phenology of prey availability, rather than contemporaneous conditions [68]. Additionally, blue whales foraged in locations that were highly predictable year-to-year and had high long-term productivity. These results suggest blue whales use memory to time their northward migrations to exploit shifting hotspots of predictable and high-quality resources. Blue whales are thought to maximize resource gain during migration by targeting predictable foraging areas, a strategy that should favour memory [69,70] (see electronic supplementary material for further details). How extensively migratory behaviour is influenced by social learning and/or individual experiences is unclear. However, blue whales likely have capacity for social learning; blue whales sing population-specific songs (creating vocal dialects), while new research is teasing apart the influence of social learning in their migratory and vocal behaviour [71–73].

## 4. Vocal dialects and song culture

### (a) Humpback whale song culture

The songs of humpback whales are recognized as one of the strongest and clearest cases for social learning and resultant vocal culture in any animal species [5,7,23,24]. Like many other baleen whales, humpback whales migrate annually between high latitude summer feeding areas and low latitude winter breeding areas (e.g. [74,75]). On migration as well as on the breeding grounds, male humpback whales produce complex, highly structured songs primarily during the breeding season [76,77] (see electronic supplementary material for further details). The song is also shared among all the males in any particular population, so that males use the same sounds arranged in similar ways. Although the song’s pattern changes within the population over time, all or most singers update their songs to maintain concurrent song matching [19]. The rate of song change varies, but large-scale changes or ‘evolutions’ in songs generally occur over a few years. ‘Revolutionary’ song changes, where entire new songs from a neighbouring population can enter and spread through a population, may occur over as little as a few months within a single singing season [23]. Song revolutions that sweep across an ocean basin in a series of cultural waves and shared song among different ocean basins occur within Southern Hemisphere populations [24], where the open Southern Ocean allows for mixing of individuals from different breeding populations on the feeding grounds [78,79]. This likely allows for song transmission between populations either via changes to individual migratory destinations or through shared migratory corridors as whales leave the feeding areas [80,81]. Most records of song revolutions come from the South Pacific Ocean [24,82,83], but recently a potential song revolution was documented in the South Atlantic (Brazil) [84,85]. This supports evidence for a ‘Southern Ocean Exchange’ [79,80], which would allow a full circumpolar exchange of song. Thus far, there is no evidence of song revolutions occurring in the Northern Hemisphere where such circumpolar interchange is not possible [81]. Although the process itself is largely hidden by the difficulty of studying individual whales over the necessary time spans, the products of this process, the songs themselves, tell a startlingly clear story of social learning and emergent culture, with each population having at any time its own population-wide vocal display.

### (b) Acoustic behaviour in the bowhead whale

Among baleen whales, only humpback and bowhead whales (*Balaena mysticetus*) sing complex songs that change within and between years in a population. Like humpback whales, bowhead whales sing seasonally, and it is presumed to be a male display, although this remains unconfirmed. However, unlike humpback whales, bowhead whales sing dozens of distinctly different song types within a single breeding season, and this behaviour occurs under sea ice during the polar night (i.e. during the boreal winter) [86] (see electronic supplementary material for further details). Data from multiple years and two populations of bowhead whales suggest that the notes used to compose songs change completely between years, driving song variability [86,87]. The recording of overlapping, distinctly different songs suggests that not all individuals in a population sing the same song at the same time [88,89]. While some songs only persist for days, others can persist for weeks or even months, being sung by multiple individuals and providing evidence that these are truly shared, socially learned songs within a population and not just individually distinct vocal patterns [86]. Preliminary evidence also suggests that some whales produce very similar songs, which are recorded close in time (within days to at most weeks), while others do not [90]. What has yet to be explored is whether population-level differences in singing behaviour or song composition exist in the four different, geographically isolated populations of bowhead whales. The great level of flexibility in bowhead whale acoustic behaviour combined with their 150−200 years lifespan [91] indicates a capacity for learning and adaptation. This suggests that these variations in acoustic behaviour might represent a form of culture [92].

## 5. Key indicators and conservation implications of social learning

Climate change and other anthropogenic impacts have placed many ecosystems and the animals within them under great pressure. In times of rapid change, adaptability is vital, and for any species, adaptation relies on both genetic and behavioural variation, as well as epigenetic, phenotypic and behavioural plasticity. While most conservation efforts to date have focused on preserving variation through genetics, another major source of variability for a species is via socially learned behaviours. These can vary in scale from familial groups to entire populations, where they can result in cultures. Such heterogeneity may create differences in vital rates and fitness of individuals within and/or between populations [15,93]. Understanding the interplay between social learning, behavioural plasticity and the environment is key [94]. The above case studies clearly show the likely importance of social learning in foraging, migration and vocal behaviour for baleen whales. Social learning may also be supported by other traits, such as memory (e.g. blue whale [68]; humpback [81]) and communication (e.g. feeding call facilitating coordination in humpbacks). We now turn to the available evidence in the literature as to whether social learning could influence these behavioural contexts in other baleen whale species (table 1). Here, we follow the framework proposed by Brakes *et al*. [6,27], which considers indirect or correlative evidence for social learning (Panel 1), but also suggests that in the absence of such evidence, researchers can consider whether there might be opportunities in the life history of these animals where social learning might occur. We use expert opinion to assess the strength of this evidence for whether conservation decisions should consider social learning (table 2). We integrate this evidence with IUCN Red List status [113] for each baleen whale species to support Panel 3 of Brakes *et al*.’s [6,27] practical framework where future conservation assessment and management could include social learning and culture.

**Table 1.** Potential indicators of social learning in baleen whales and how such learning might interface with various behavioural contexts. (a) Does current evidence suggest or support the potential for a role of social learning by major behavioural contexts? Yes (Y), no (N), no evidence (NE) or unknown (UN). Evidence for potential social learning can be vertical (V), maternally directed (MD) (a special case within vertical), oblique (O), horizontal (H) or unknown (UN). ? = Expert opinion of suggestive but unclear evidence. (b) Is there variation in any of the behavioural contexts within or between populations? Yes (Y), no (N) or unknown (UN). Are any of these variations persistent? (timescale: days, weeks, months, years, decades, centuries). n/a = Single population present. Popn = population.

species	common name	indicators of social learning/culture			variation and persistence of behaviour				potential for social learning	references
		migration	foraging	vocal	variation behavioural trait *within* popn	is this persistent?	variation behavioural trait *between* popn	is this persistent?		
Balaenidae										
*Balaena mysticetus*	Bowhead whale	Y (MD)	Y (H?)	Y (H?)	Y (song, migration)	Y (migration; centuries) N (song; weeks)	Y (migration, song?)	Y (migration; decades)	Y (MD, H?)	[86,95,96]
*Eubalaena australis*	Southern right whale	Y (MD)	Y (H)	NE	Y (migration, foraging)	Y (centuries)	Y (migration)	Y (centuries)	Y (MD, H)	[16,97]
*Eubalaena glacialis*	North Atlantic right whale	Y (MD, H)	NE	Y (UN)	Y (migration)	Y (centuries)	n/a	n/a	Y (MD, H)	[62,63,98]
*Eubalaena japonica*	North Pacific right whale	UN	UN	UN	UN (song?)	UN	UN	UN	UN	[99]
Balaenopteridae										
*Balaenoptera acutorostrata*	Common minke whale	Y (MD)	Y (UN)	Y (UN)	Y (foraging, song)	Y (UN)	Y (migration, song)	Y (UN)	Y (MD)	[100,101]
*Balaenoptera bonaerensis*	Antarctic minke	Y (UN)	Y (UN)	Y (H?)	UN	UN	UN	UN	UN	[102,103]
*Balaenoptera borealis*	Sei whale	Y (MD)	UN	Y (UN)	Y (migration)	Y (years)	UN	UN	Y (MD)	[104,105]
*Balaenoptera edeni* spp.	Bryde’s whales	Y (MD)	Y (MD, H)	UN	Y (foraging)	Y (decades)	Y (migration, foraging)	Y (decades)	Y (MD, H)	[46–48,106]
*Balaenoptera musculus* spp.	Blue whale	Y (MD?, H)	UN	Y (H)	Y (song)	Y (decades)	Y (song)	Y (decades)	Y (MD?, H)	[68,71,73]
*Balaenoptera omurai*	Omura’s whale	UN	UN	Y (H)	Y (song)	UN	Y (song)	UN	Y (H)	[107]
*Balaenoptera physalus*	Fin whale	Y (MD?)	UN	Y (H)	Y (migration)	Y (UN)	Y (migration, song)	Y (decades)	Y (MD?, H)	[72,108,109]
*Balaenoptera ricei*	Rice’s whale	N	UN	NE	N	N	n/a	n/a	UN	
*Megaptera novaeangliae*	Humpback whale	Y (MD, H)	Y (H)	Y (H)	Y (foraging, migration, song)	Y (foraging, migration; decades) N (song; 1−2 years)	Y (foraging, migration, song)	Y (decades)	Y (MD, H)	[17,18,24,79,84,85]
Neobalaenidae										
*Caperea marginata*	Pygmy right whale	UN	UN	UN	UN	UN	UN	UN	UN	
Eschrichtiidae										
*Eschrichtius robustus*	Gray whale	Y (MD, H)	NE	NE	Y (migration)	Y (centuries)	Y (migration)	Y (centuries)	Y (MD, H)	[110–112]

**Table 2.** Summaries of the evidence for social learning (and culture) from table 1 listed by IUCN Red List classification status [113]. Multiple listings may be present if there are IUCN, IWC or nationally recognized subpopulations: Critically Endangered (CR); Endangered (EN); Vulnerable (VU); Near Threatened (NT); Least Concern (LC); Data Deficient (DD). Weight of evidence: (S) strong: requires a peer-reviewed publication of this evidence (in table 1); (W) weak: either weak direct evidence of social learning, or social learning likely but this is through inference of evidence from closely related species (e.g., within the right whales); (NE) no evidence: research has been undertaken but no evidence of social learning found; (UN) unknown: no available research. Expert opinion was used to categorize the strength of this evidence (based on references from table 1) for whether conservation decisions should consider social learning (and culture) for each behavioural context.

IUCN Red List status	species	common name	strength of evidence for inclusion in conservation decisions		
			migration	foraging	vocal dialects/song
CR	*Eubalaena glacialis*	North Atlantic right whale	S	NE	W
CR	*Balaenoptera ricei*	Rice’s whale	NE	UN	NE
EN	*Eubalaena japonica*	North Pacific right whale	UN	UN	UN
EN	*Balaenoptera borealis*	Sei whale	W	UN	W
EN	*Balaenoptera musculus* spp*.*	Blue whale	S	UN	S
VU	*Balaenoptera physalus*	Fin whale	W	UN	W
NT	*Balaenoptera bonaerensis*	Antarctic minke	W	W	W
LC (EN: sub-popns)	*Eubalaena australis*	Southern right whale	S	W	NE
LC (EN: sub-popns)	*Megaptera novaeangliae*	Humpback whale	S	S	S
LC	*Balaena mysticetus*	Bowhead whale	S	W	S
LC	*Balaenoptera acutorostrata*	Common minke whale	S	W	W
LC	*Balaenoptera edeni* spp*.*	Bryde’s whales	S	S	UN
LC	*Caperea marginata*	Pygmy right whale	UN	UN	UN
LC	*Eschrichtius robustus*	Gray whale	S	NE	NE
DD	*Balaenoptera omurai*	Omura’s whale	UN	UN	W

In the context of foraging behaviours, humpback and Bryde’s whales illustrate where the intersection between social learning and conservation can occur given the high degree of plasticity in their foraging tactics [37]. Humpback whales appear to adapt and innovate their tactics in response to surrounding environmental conditions. Instances of bubble feeding in the Gulf of Maine follow the movement of mobile prey patches [114], while lunge feeding rates decline with water temperature on the east Antarctic feeding grounds [115]. Social learning is likely to be a key factor in the ability of humpback whales to respond and adapt to environmental change. Furthermore, since these responses often involve specialization, they could have significant downstream ecological effects on targeted prey. While not highly social whales, Bryde’s whales may aggregate around prey patches where behavioural specializations may be transmitted either through direct observation of other adults or passed from mothers to calves. The apparently high level of local specialization in behaviours globally may make Bryde’s whale populations vulnerable to disturbance. For populations where individuals switch prey types and foraging strategies, these may be a suite of learned behaviours. Capacity for memory in rorquals, demonstrated by work on blue whales, highlights how such a repertoire could be maintained. The importance of foraging culture in humpback and Bryde’s whales and its relationship with both their ecology and their environment emphasizes the need to understand the role of foraging culture across baleen whale species. Foraging plasticity and behavioural change within a population can be seen as strong potential indicators of social learning along with consistent differences between populations (table 1).

In the context of migratory traditions, the case studies show that we see fidelity to migratory terminals that is likely socially learned, as well as variation when individuals move to other areas (table 1) [93]. Cultural knowledge of migratory habitats and shifting habitat use in response to climate change illustrates a mix of behavioural conservancy and plasticity. This mix appears to shape recovery from exploitation in both southern right whales [65] and to some extent North Atlantic right whales [60–64]. In the case of southern right whales, this is on a circumpolar scale allowing large-scale processes to be gleaned. For example, southern right whales have ‘rediscovered’ some previous migratory destinations (e.g. mainland New Zealand and Falkland Islands [32,116]). These events have occurred over nearly a century, whereas conservation measures focus on shorter timescales. This suggests that protecting key migratory habitat and the whales that use them is essential to maintaining a range of migratory traditions in a population. Both maternally directed learning of migratory destinations and long-term site fidelity in *Eubalaena* are also present in humpback whales [18], and are likely to be shared with other baleen whale species. Furthermore, *Eubalaena* also display some behavioural flexibility, shown by their shift in foraging grounds in response to changing prey abundance. The mechanism(s) underpinning such shifts are still poorly understood. As demonstrated in the blue whale case study, memory and individual experience likely play a role, but the ability to socially learn new migratory routes is also a potential mechanism that has not been fully investigated. Social learning can thus be a conservative force encouraging animals to follow their mothers at the risk of overlooking good habitat, a way for adults to update or modify their knowledge of good foraging sites, and a way of teaching young animals to use newly ‘rediscovered’ habitats (and potentially starting a new tradition).

Finally, shared, rapidly changing song dialects are strong indicators of social learning in the context of vocal communication (table 1). Both humpback and bowhead whales display vocal plasticity and rapid change that cannot be explained without social learning. Other baleen whale species display population-level song dialects composed of less complex vocal repertoires. This provides weaker evidence for social learning contributing to vocal behaviour(s) where song has been described. Social calls have been less well studied in most species but may also provide evidence of dialects. Whether or not song patterns are adaptive is unknown. Although ephemeral in humpback and bowhead whales, if these song patterns provide some indication of fitness, they may have an important role in the mating system. A major conservation concern is ocean noise (e.g. shipping, seismic and sonar) which can mask or interrupt vocalizations [117–122]. The location and timing of such activities (e.g. on a breeding ground during the breeding season) clearly need to be considered, along with ongoing efforts to quantify population-level consequences of such disturbances.

Culture can play an important role in cetaceans’ lives [7], with multiple, independently evolving traditions from different behavioural contexts able to be present within a species or population [17]. Given that there is increasing recognition of social learning and culture as important factors that may impact the viability and structure of populations and influence animals’ responses to conservation strategies [5,6], we have provided a guide to identify key potential indicators of social learning in baleen whales. This effort is in support of the work conducted by the Expert Group on Animal Culture and Social Complexity to embed animal culture into the conservation initiatives of the CMS, an international treaty operating under UNEP. We hope this effort will assist both scientists and conservation practitioners along with policy makers in embedding animal culture and social learning into conservation policy and practice in the years to come.

## Data Availability

Supplementary material is available online [123].
